# Are elder siblings helpers or competitors? Antagonistic fitness effects of sibling interactions in humans

**DOI:** 10.1098/rspb.2012.2313

**Published:** 2013-01-07

**Authors:** Aïda Nitsch, Charlotte Faurie, Virpi Lummaa

**Affiliations:** 1 Department of Animal and Plant Sciences, University of Sheffield, Sheffield S10 2TN, UK; 2 CNRS, Institut des Sciences de l'Evolution, Place Eugène Bataillon, CC 065, 34095 Montpellier Cedex 5, France; 3 Wissenschaftskolleg zu Berlin—Institute for Advanced Study, Wallotstraße 19, 14193 Berlin, Germany

**Keywords:** family evolution, cooperative breeding, sibling rivalry, life-history trade-off, kin selection

## Abstract

Determining the fitness consequences of sibling interactions is pivotal for understanding the evolution of family living, but studies investigating them across lifetime are lacking. We used a large demographic dataset on preindustrial humans from Finland to study the effect of elder siblings on key life-history traits. The presence of elder siblings improved the chances of younger siblings surviving to sexual maturity, suggesting that despite a competition for parental resources, they may help rearing their younger siblings. After reaching sexual maturity however, same-sex elder siblings' presence was associated with reduced reproductive success in the focal individual, indicating the existence of competition among same-sex siblings. Overall, lifetime fitness was reduced by same-sex elder siblings' presence and increased by opposite-sex elder siblings' presence. Our study shows opposite effects of sibling interactions depending on the life-history stage, and highlights the need for using long-term fitness measures to understand the selection pressures acting on sibling interactions.

## 1. Introduction

Determining the importance of family settings for individual success interests scientists from several disciplines, such as developmental psychology, genetics, social sciences and evolutionary biology. Evolutionary studies of family effects on offspring growth and development have traditionally focused on parental investment and parent–offspring conflict [1–3]. However, when several young co-reside in a family, sibling relationships are also likely to have important developmental, psychological, morphological or behavioural consequences [4]. Potentially, opposite sibling interactions can occur simultaneously in a family: competition and cooperation. While sibling relationships have been studied mostly in the context of negative effects of competition to monopolize limited resources, such as parental care [2], positive effects of siblings can also arise, for example when elder siblings help to raise their younger siblings by providing food or protection [5]. Both are widespread across the most studied taxa in this respect, namely insects, birds and mammals [6–8], with the prevailing strategy depending on factors within a family (e.g. sex and birth order [9]), across families (e.g. number of siblings or the quality of parental territory [4,10]) and finally, between populations or species (e.g. life histories or population density [11,12]).

While such studies have contributed to our understanding of the importance of sibling relationships for individual growth and development [4], three important shortcomings preclude conclusions about the overall selection on family living. First, the current knowledge of sibling effects is limited only to short-term measures, such as condition, growth, offspring weight or survival to breeding age [12]. As a consequence, potential effects of siblings on other fitness outcomes, such as reproductive success, and the overall importance of sibling effects in affecting lifetime fitness are not well studied. Second, previous studies ignore the possibility that later-life sibling effects could be opposite to those identified during development [12,13] or that the optimal strategy for siblings can change across life. Such changes are feasible, because the scope for competition for parental resources, territories or breeding opportunities is likely to vary across life stages, as are the costs and benefits of cooperation [1]. Third, current studies focus mostly on birds, which limits the scope of understanding sibling relationships in a broader sense, given that, in mammals, siblings can interact already *in utero* and lactation provides a different basis for food competition [4]. These shortcomings result partly from the difficulty of collecting long-term datasets allowing evaluation of overall fitness [13] and has led to bird studies stimulating most of the theoretical work on sibling interactions [4].

In humans, short birth intervals and the relatively long dependency period of offspring imply that different-aged offspring often have to live together. Therefore, sibling interactions are also expected to have important effects on an individual's fitness [14]. To support this, several behavioural studies have suggested that elder siblings could participate either to the resource collection or act as helpers at the nest to assist in raising younger siblings, thereby potentially enhancing mother's fertility or improving sibling condition and survival ([15–17], but see Kramer [18] for a recent review).

Several detailed long-term datasets are now available for human populations that allow investigating the long-term effects of siblings' presence on individual success, as well as factors influencing them [13]. Previous studies using such data suggest that both positive and negative effects of siblings within a human family can be important. First, similar to many other species, presence of siblings can have negative effects on outcomes such as children's development (body-mass index, height or skin fold thickness) or survival [19,20]. Second, some studies have also showed that elder sisters could improve sibling condition and survival during childhood [21]. Nevertheless, these findings are not universal: a study on Dogon, traditional agriculturalists of Mali, suggested that rather than helping, siblings competed for resources, resulting in a trade-off between the number of offspring and their growth and survival [22]. However, only the total number of siblings was considered, preventing distinguishing between competition over parental resources and potential helping behaviour of elder siblings. Many previous studies have suffered from inability to consider confounding factors that could modify sibling relationships, such as birth order, sex, socio-economic status (SES), mother's survival or the total family size reflecting overall level of competition [21,23,24]. Moreover, as in most other species studied, no study has investigated overall fitness consequences of sibling relationships, leaving the net outcome from the documented negative and positive sibling effects unknown, and how such effects depend on key traits such as sex, birth order or family resources.

Our study is the first to investigate sibling effects on overall lifetime reproductive success in humans. We use a large, longitudinal, individual-based dataset of preindustrial Finns collected from parish church registers [25]. This dataset is particularly suited to identify the resulting outcome of variation in family configuration across the life stages, as it provides close estimates of individuals' fitness for complete families (five offspring per reproducing female on average) with a high offspring follow-up success (91% of individuals followed until age 15 years) [25], as opposed to most previous studies realized in contemporary populations with small datasets mostly relying on individuals' memory. Our dataset also allows investigating the role of factors potentially shaping sibling interactions both at the individual (sex and birth order) and family level (father's SES, mother's survival and sibship size) among five geographically isolated populations living in conditions close to natural fertility and mortality [25]. Although we acknowledge that the conditions experienced by preindustrial Finns are unlikely to resemble those of our hominin ancestors and the importance of sibling interactions may also vary widely across different socio-cultures and demographic settings, our data offer a rare opportunity to investigate the outcomes of sibling interactions and family configuration using people with natural fertility and mortality competing for ecologically scarce resources [26,27], while controlling for confounding key factors such as SES.

Specifically, we investigated (i) the effects of the number of elder brothers and sisters on the survival of their younger siblings to sexual maturity (age 15 years); (ii) the effects of the number of elder brothers and sisters on two measures of lifetime reproductive success: the probability of reproducing in a lifetime and the lifetime fecundity of those producing at least one offspring; (iii) whether age at first reproduction or SES was associated with the number of elder brothers and sisters, and the effect of primogeniture (first offspring inheritance of wealth); and (iv) the net outcome of elder siblings on lifetime fitness by combining their effects during childhood (on survival to sexual maturity) and adulthood (on reproductive success).

## 2. Material and methods

### (a) Study population

The demographic dataset from historical Finnish populations was compiled from records of the Lutheran church, which was obliged by law to document all dates of births, marriages and deaths in the population for tax purposes [25–29]. As migration events were relatively rare and the migration records maintained by the church allowed us to follow dispersers in the majority of the cases, these records provide us with relatively accurate information on individual survival and reproductive histories [28] (e.g. 91% of individuals with known birth date were followed to sexual maturity at age 15 years). Our study period is limited to the eighteenth and nineteenth centuries, before the transition to reduced birth and mortality rates [30]. We included five geographically distinct parishes into our analyses, which depended on farming and fishing for their livelihood (Ikaalinen, Tyrvää, Kustavi, Rymättylä and Hiittinen). We categorized all individuals into three SES groups (treated as a three-level categorical variable in the analyses) according to the occupation for adult men (husband's occupation for women): low (e.g. farmless families and servants), middle (e.g. tenant farmers, smiths and fishermen) and high (e.g. aristocrats and landowners). Distinguishing these different categories of resource availability was important as SES was significantly associated with survival, reproductive success and selection on different life-history traits in Finnish populations [28,31], and it has been shown that sibling interactions could depend on SES in this population [29] or other agricultural populations [32]. Overall, the standard of living was low with both famines and diseases common [33]. The main causes of death were infectious diseases associated with malnourishment [33]. Mortality rates were high, especially among children: more than 30 per cent did not reach maturity (15 years of age, the youngest known reproducer in our population) [28]. Surviving offspring usually moved away from home to work from around age 15 years onwards, but commonly returned home [34]. Similar to the general European pattern at the time, the average age at first marriage was 25 and 27 years for women and men, respectively (see [35]), and 76 per cent of individuals in the sample married if they survived to age 15 years. Inheritance usually favoured the eldest son (primogeniture), and the first daughter inherited a higher dowry (and the majority of wealth in the case of no male heir) [36]. The typical household was composed of the eldest son, his wife, their children, his parents and one or more unmarried siblings. All siblings usually lived close by [34]. The mating system was patrilocal and monogamous; divorce was forbidden [34].

The study sample contains 10 106 focal males and 9585 focal females born 1750–1900 to 3829 mothers, and all of their 29 385 offspring born 1770–1958. The sample is restricted to individuals for whom the variables included in our statistical analysis (see below) were available (77% of the overall sample). Twins (4%) were excluded due to their lower survival [37].

### (b) Statistical analyses

All statistical analyses were conducted on R software v. 2.11.1 [38] using generalized linear mixed effects models (GLMMs; function *lmer*, *lme4* package [39]). We conducted all analyses separately for each sex because of differences between the sexes in parental investment and life histories [29,40].

Two variables of interest were included in the analyses of survival or reproductive success measures: the numbers of elder sisters and brothers alive at the beginning of each study period (at focal individual's birth, or at age 15 years, see below), fitted as independent continuous variables (those with four or more elder brothers or sisters were pooled to four, to avoid influence of individuals with extreme numbers of elder brothers and sisters). These two variables were first included in models as both quadratic and linear terms, but as the quadratic terms were non-significant, final models only include the linear term. We focused on the number of elder siblings because in humans they are expected to exhibit more cooperative breeding behaviour during childhood [21]. Unlike several previous studies investigating the potential care provided by elder siblings, we considered the effects of all elder siblings and not only those at least 3 years older than their younger siblings [20,41]. This was because we investigated the effects of siblings across the life of the focal individual rather than only during childhood, and because limiting the sample to only those siblings an arbitrary number of years older than the focal individual could bias the sample to laterborn children [21].

In all analyses, parish, birth year and mother's identity were fitted as random effects to account for the dependency owing to shared family, the same geographical area or the same year. Because random effects are generally better estimated with at least three observations for each level of the random term [42], we excluded families of less than three children. The significance of each term was tested with likelihood ratio tests comparing the full model to those without the term of interest. All potential two-way interactions between the number of elder sisters or brothers and other variables were initially tested, but removed if non-significant at the level of *α* = 0.05.

#### (i) Survival to adulthood

First, we investigated the effect of elder brothers and sisters on the survival to sexual maturity of the focal individuals. Survival to age 15 years was scored as a binary response (0 = did not survive, 1 = survived), and analysed using GLMMs with a binomial error structure and a logit link function. This analysis was restricted to individuals followed successfully until age 15 years (10 106 males and 9585 females). Each model included as fixed effects mother's age, father's SES (included as a three-level categorical variable), mother's survival (mother alive or dead at the end of childhood), birth order (firstborn or laterborn to account for lower survival of firstborns in the study population) [29] and the total number of siblings (to control for overall competition for resources in the family). Total number of siblings included the number of elder siblings alive at birth and the number of younger siblings born during the study period (birth to age 15 years of focal individual).

#### (ii) Reproductive success

Second, we investigated the long-term effects of elder siblings on reproductive success. The sample was restricted to individuals who survived until sexual maturity (age 15 years) and who were successfully followed to death or at least until the age when 90 per cent of individuals in the population had ceased reproduction (50 and 45 years for males and females, respectively). This sample included 3201 males and 3292 females. Because of the high number of individuals who never reproduced and methods allowing the inclusion of a large number of zeros in GLMMs being poorly developed [43], reproductive success was analysed in two steps: (i) the probability of reproducing, and (ii) the total number of offspring born to those individuals who had at least one child (2104 males and 2459 females). Potential confounding factors fitted as fixed effects included the total number of siblings (alive at age 15 years), the father's SES and the age of an individual at mother's death. *Probability of reproducing* was analysed with a binomial error structure and a logit link function*. Lifetime fecundity* was analysed with a Poisson error structure and a logarithm link function.

#### (iii) Underlying life-history traits

Our approach tested the correlation between the presence of elder siblings and the fitness of younger siblings, which did not allow us to determine the underlying mechanisms of these correlations. However, we investigated the relevance of some variables potentially mediating these effects.

First, to test whether the association between offspring count and number of elder siblings was partly mediated by an effect of elder siblings on age at first reproduction, we added this latter variable to the previous model on lifetime fecundity. Second, we tested an effect of primogeniture on the detected results. When the effect of being the heir (a son/daughter with no elder brothers/sisters alive at age 15 years) was significant, we tested separately the effect of elder brothers and sisters in a subsample including only non-heir individuals. Third, to test whether elder siblings had an effect on the SES individuals achieved in adulthood, we fitted GLMMs with a binomial error structure and a logit link function to the adulthood probability of owning land. Landless individuals included those who either rented land (tenant farmers) or worked as servants (i.e. middle or low SES). The fixed and random effects included were the same as in previous models.

#### (iv) Combined sibling effects on lifetime fitness

In order to investigate the net effect of elder siblings on lifetime fitness, we combined the effect of elder siblings on survival to sexual maturity and on reproductive success to estimate the lifetime fecundity expected at birth. This latter variable was estimated by multiplying the predicted probability of surviving to age 15 years by the probability of reproducing and by the expected offspring count, for all possible numbers of elder brothers and elder sisters, obtained from the previous models. Other continuous covariates were fixed to their mean value. Categorical covariates were arbitrarily fixed to a specific level, as no interactions were significant in the models: high for family SES, laterborn for firstborn status and alive for mother's survival during childhood.

## 3. Results

### (a) Sibling effects in childhood

#### (i) Males

**Figure 1.**
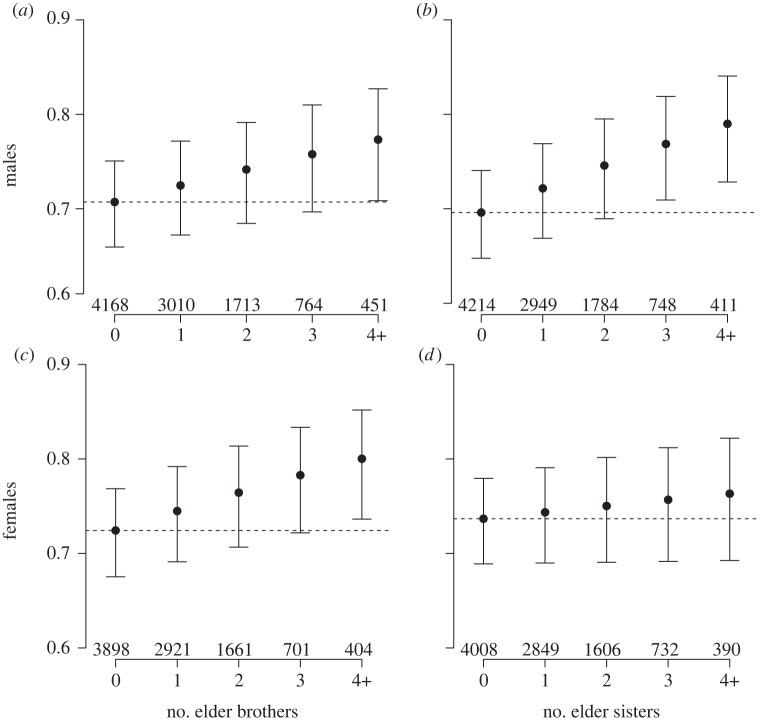
Probability of surviving to age 15 years: (*a*) of males according to their number of elder brothers (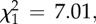 *p* = 0.008). (*b*) of males according to their number of elder sisters (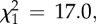 *p* < 0.001). (*c*) of females according to their number of elder brothers (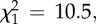 *p* = 0.001). (*d*) of females according to their number of elder sisters (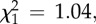 *p* = 0.31). Figures show predicted values of the model and error bars represent standard errors of the means. The horizontal dashed line represents the predicted value in the case where the individual had no elder brothers or no elder sisters. Numbers below bars represent the sample size.

Overall, 66.3 per cent of all males (*n* = 10 106) survived to age 15 years. Elder brothers and sisters had similar effects on their younger brother's survival to adulthood. Each additional elder sister (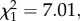 *p* = 0.008) and elder brother (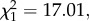 *p* < 0.0001) alive at the younger brother's birth was associated with a 1.12 (confidence interval, CI 95% = 1.07–1.20) and 1.09 times increase (95% CI = 1.03–1.15), respectively, in his probability of surviving to age 15 years (figure 1*a,b*; electronic supplementary material, table S1*a*). These findings were not confounded by differential maternal survival, maternal age, family SES or overall level of within-family sibling competition resulting from differences in the total number of siblings, which were all controlled for.

#### (ii) Females

On average, 68.1 per cent of all females (*n* = 9585) survived to age 15 years (figure 1*c*,*d*; electronic supplementary material, table S1*b*). Each additional elder brother alive at the younger sister's birth increased her probability of surviving 1.11 times (95% CI: 1.04–1.18, 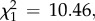 *p* = 0.001), whereas no significant effect of elder sisters was found (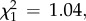 *p* = 0.31). This model controlled for the effect of maternal survival, maternal age, sibship size and family SES.

### (b) Sibling effects in adulthood

#### (i) Males

Overall, we found no indication of beneficial effects of siblings on male reproductive success in adulthood, but instead there was evidence of same-sex competition.

**Figure 2.**
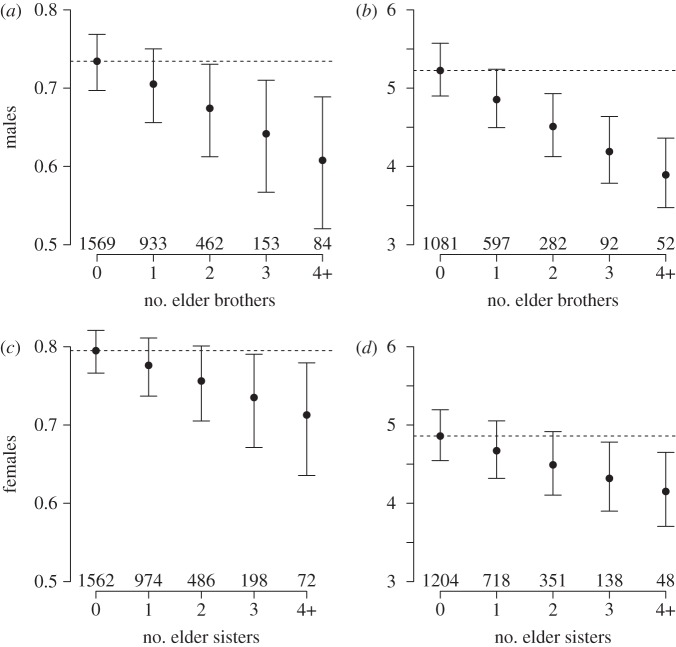
Reproductive success of individuals surviving to adulthood: (*a*) Males' probability of reproducing (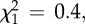 *p* < 0.001) and, for those who reproduced, (*b*) offspring count (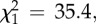 *p* < 0.001), according to their number of elder brothers. (*c*) Females’ probability of reproducing (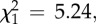 *p* = 0.02) and, for those who reproduced, (*d*) offspring count (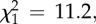 *p* < 0.001), according to their number of elder sisters. Figures show predicted values of the model and error bars represent standard errors of the means. The horizontal dashed line represents the predicted value in the case when the individual had no elder brothers or no elder sisters. Numbers below bars represent the sample size.

First, 65.7 per cent of males (*n* = 3201) who survived to adulthood reproduced in their lifetime, and each additional elder brother alive when the younger brother reached adulthood decreased this probability of ever reproducing (odds ratio, OR = 0.87, 95% CI = 0.80–0.96, 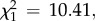 *p* = 0.001), whereas there was no effect of elder sisters (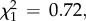 *p* = 0.40; figure 2*a*; electronic supplementary material, table S2*a*). The model controlled for significant effect of family SES.

Second, the lifetime number of children (5.4 ± 0.07 s.e.) among males who reproduced at least once (*n* = 2104) was also negatively associated with their number of elder same-sex siblings alive at the onset of adulthood (*β* = −0.07 ± 0.01 s.e., 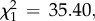 *p* < 0.0001), whereas elder sisters had no significant effect on a male's number of children (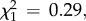 *p* = 0.59; figure 2*b*; electronic supplementary material, table S3*a*). The model controlled for significant positive effects of the overall number of siblings.

When including age at first reproduction (on average 28.38 ± 0.13 s.e.) in the previous model, we found that it was, as expected, negatively associated with a male's lifetime number of children (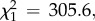 *p* < 0.001). The negative effect of elder brothers was, however, still significant (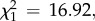 *p* < 0.001), but its magnitude was decreased, suggesting that its effect on lifetime number of children was partly mediated by an effect on age at first reproduction (*β* = −0.05 ± 0.01 s.e., 29% decrease). This model controlled for significant effect of the sibship size (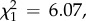 *p* = 0.014) and age at mother's death (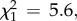 *p* = 0.018).

Another mediating factor may be that the probability of a son becoming a landowner himself in adulthood was negatively associated with the number of elder brothers among landowning families (SES × number of elder brothers: 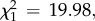 *p* < 0.001). We consequently also investigated a potential primogeniture effect in males, and found that the heir of the family had a higher probability of reproducing (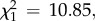 *p* = 0.001) and a higher number of children than his younger brothers (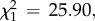 *p* < 0.001). When considering a smaller sample of only non-heirs (*n* = 2144), the effect of elder brothers was non-significant on the probability of reproducing (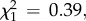 *p* = 0.57), but remained significant and negative on the number of children born (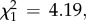 *p* = 0.04) although the effect magnitude was lower (*β* = −0.04 ± 0.02 s.e., 43% decrease).

#### (ii) Females

Similar to males, we found no indication of beneficial effects of siblings on female reproductive success in adulthood, but there was again evidence of same-sex competition.

First, 74.7 per cent of females who survived to adulthood reproduced at least once (*n* = 3292), and this probability was negatively correlated with the number of elder same-sexed siblings (sisters) alive when the younger sister reached adulthood (OR = 0.90, 95% CI = 0.82–0.98, 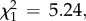 *p* = 0.02), but was not significantly associated with the number of elder brothers (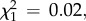 *p* = 0.88; figure 2*c*; electronic supplementary material; table S2*b*).

Second, each elder sister alive at adulthood decreased their sisters' number of children (5.03 ± 0.06 s.e.) among those females who reproduced at least once (*n* = 2459, *β* = −0.04 ± 0.01, 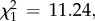 *p* = 0.001), whereas the number of elder brothers had no effect on lifetime fecundity (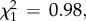 *p* = 0.32; figure 2*d*; electronic supplementary material, table S3*b*). The model controlled for significant effects of family SES.

Such effects might arise partly from females with elder sisters being less likely to ever marry (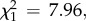 *p* = 0.005). Nevertheless, when adding age at first reproduction (25.8 ± 0.10 s.e.; 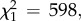 *p* < 0.001) to the previous model on lifetime fecundity, the negative effect of elder sisters remained significant (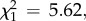 *p* = 0.02; *β* = −0.03 ± 0.01 s.e., 25% decrease). The number of elder sisters was further negatively associated with the probability of marrying a landowning man (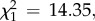 *p* < 0.001), whereas the number of elder brothers was not (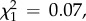 *p* = 0.8). This model controlled for the significant effect of the mother's age (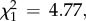 *p* = 0.03) and the higher overall probability of marrying a landowner among daughters of landowner fathers (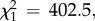 *p* < 0.001).

### (c) Combined sibling effects on lifetime fitness

To evaluate the overall importance of any detected sibling effects across lifetime on overall fitness, we incorporated any effects of siblings (positive or negative) on chances of surviving, of reproducing and on lifetime fecundity into one single outcome (at-birth lifetime fecundity).

For males, the negative effect of elder brothers on reproductive success during adulthood outweighed their positive effect on survival during childhood, and resulted in a negative overall effect of elder brothers on their younger brothers' lifetime measure of fitness. By contrast, the positive effect of elder sisters during childhood was translated into a higher overall success (see the electronic supplementary material, table S4*a*). The lifetime measure of fitness of females was positively associated with the presence of elder brothers and negatively with the presence of elder sisters. Therefore, the positive influence of the presence of elder brothers on females' survival translated into higher at-birth fecundity, and the negative influence of the presence of elder sisters on female reproductive success induced lower lifetime fitness (see the electronic supplementary material, table S4*b*).

## 4. Discussion

Understanding the importance of sibling interactions for individual fitness has been limited by the lack of studies investigating sibling effects across the whole lifespan. Our study is the first to provide a comprehensive view of the influence of sibship configuration on fitness outcomes, and to investigate both negative effects (owing to competition for parental resources) and positive effects (owing to kin selection processes). Our results document the interactions between elder and younger siblings' sexes, a topic rarely examined despite previous studies suggesting both intra- and inter-sex competition [29,44,45]. Our study highlights (i) a positive association between the number of elder siblings and children's survival to adulthood, (ii) a negative association between the number of same-sex elder siblings and several components of reproductive success; and (iii) overall, for a measure of lifetime fitness, a positive association with the number of opposite-sex elder siblings and a negative association with the number of same-sex elder siblings.

Our finding that both positive and negative sibling effects can occur on different fitness components, and at different life stages, implies that optimal trade-offs between competitive and cooperative behaviour could vary across the lifespan. Few previous studies have investigated opposing effects of siblings on fitness outcomes at different stages, but one study on red wolves (*Canis rufus*) suggested that helpers were beneficial for both male and female younger siblings' juvenile survival, but negatively associated with male and positively associated with female reproductive success [12]. Our study is the first to reveal, in humans, such opposing associations between sibling presence and stage-specific fitness measures and the overall outcome of such stage-specific effects.

Our results concerning survival to reproductive maturity suggest that elder brothers and sisters helped to raise their younger siblings, thereby improving their inclusive fitness. This finding is of particular importance as survival to sexual maturity is a key determinant of fitness in this and many other traditional populations where almost half of individuals do not reach adulthood [28,46], as well as in other species [13]. The fact that elder brothers had a positive effect on both younger sisters and brothers, whereas elder sisters only had a positive effect on brothers could be due to differential sex roles, which could imply either sex-specific helping behaviour, or sex-specific competition, or both. In the study population, offspring typically stayed at least until their teens in the parental household and participated in various tasks [34]. Given boys usually worked at the farm, their participation could have increased the overall resources of the family and benefited all younger siblings independently of their sex. By contrast, girls had the opportunity to bias their help towards males as they provided direct care to their younger siblings at home. Sexual dimorphism in helping behaviour has also been reported in other human populations [20,41] and other species (e.g. Seychelles warblers *Acrocephalus sechellensis* [6] and banded mongooses *Mungos mungo* [47]), implying differential costs and benefits of cooperation and competition between the sexes. In the banded mongoose, costs of helping were higher for females than for males, explaining why caring behaviour was mostly provided by males [47]. Our results could also suggest that competition among sisters was higher than competition between sisters and brothers. The same pattern is observed when considering the effects of elder siblings on individuals, including those who did not reproduce: same-sex siblings and opposite-sex siblings are, respectively, negatively and positively associated with lifetime fecundity. This could be due to same-sex siblings competing for sex-specific resources in addition to non sex-specific family resources [48], so that within-sex competition is expected to be higher than between-sex competition.

In regression analyses of demographic datasets, the causal relations are difficult to establish given the lack of detailed information on the underlying mechanisms [9]. Therefore, the positive associations between number of elder siblings and younger siblings' childhood survival could also be interpreted as negative effects of younger siblings on their elder siblings: although the overall negative effect of sibship size was controlled for in all analyses, younger siblings could have an additional negative effect, for example if parents allocated more time and resources to the youngest. We consider this unlikely, however, because positive associations between direct measures of care or participation in resource gathering by elder siblings and younger siblings' condition have been documented in other populations [15–18], and historical records on the Finnish population describe elder siblings' helping behaviour in the household [34]. Another alternative interpretation is that parents bias their investment towards their youngest children, leading to a higher survival of laterborns. Parental investment and sibling interactions are closely connected (e.g. parents can adjust their investment according to offspring behaviour and sibling interactions are constrained by the division of parental investment) [9,18]. Nevertheless, both theoretical and empirical studies suggest that optimal parental strategies favour earlier-borns over laterborns in humans and other species [49,50], reducing the likelihood that the associations between elder sibling presence and younger sibling survival arise from parental preferences only. Note that firstborns had reduced survival during infancy [29], but that this effect was controlled for.

Our results show that among both males and females surviving until sexual maturity, reproductive success was negatively associated with the number of same-sex elder siblings. In males, the association was partly mediated by a delayed reproduction in younger brothers, caused by their later marriage (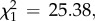 *p* < 0.0001). A larger number of elder brothers was also associated with lower reproductive success and later marriage among nomadic [44] and agropastoral African men [45]. Our results provide the first evidence of significant differential reproductive success among sisters (for non-significant similar trends, see Mace [45]). This indicates an unequal distribution of sex-specific resources, such as mating opportunities and/or parental resources that are necessary for marrying. This could be due to differential competitive abilities and/or parental strategies with differential allocation of resources. Such differential reproductive success among same-sex siblings according to birth order has also been observed among common European adder (*Vipera berus*) brothers [51].

In our study population, marriages resulted not only from individual mate preferences, but also from parental decisions and social pressures. The eldest son inherited most parental wealth (including the farm if they owned one), whereas the eldest daughter could inherit the farm only if there was no son [36]. An advantage of the primogeniture inheritance system is that as the age difference between parents and firstborns is smaller than that between parents and laterborns, favouring the reproductive success of firstborns would allow shorter generation times [50,52]. Moreover, models on the optimal strategy of investment by parents predict that favouring older offspring was the best evolutionary strategy in almost all situations [50]. In line with this statement, we highlighted that in this population firstborns of both sexes started reproduction themselves on average at a younger age than laterborns. In other agricultural populations, elder brothers also tend to reproduce earlier than laterborns [44,45]. In expanding populations such as the one studied, shortening the generation time can bring an evolutionary advantage [53]. Another advantage of shorter generation time is that grandparents are more likely to still be alive at their grandchildren's birth, enabling them to provide care to their grandchildren. This could be particularly relevant in this population, given the previous evidence of positive effects of grandmothers on offspring survival during childhood [54]. We found that the negative association between reproductive success and number of same-sex elder siblings was mostly due to primogeniture for males. However, in addition to this, we still found, among non-heirs, a negative association between number of elder brothers and the probability of reproducing. This general decrease in probability of reproducing with increasing birth order among males suggests resource dilution across time. The negative association between reproductive success and the number of same-sex elder siblings was mostly driven by a negative effect on individual SES (land ownership). For males born to landowner families, we found a negative association between the probability of becoming a landowner and the number of elder brothers. For females, such a negative association between the number of elder sisters and the husband's SES was also found, regardless of whether their parents owned land or not. This is in line with the competition over land inheritance generally concerning males, whereas females rather competed over the dowry, and were more likely to improve their social status by marrying men of higher social class. Nevertheless, when controlling for adulthood SES, the number of same-sex elder siblings was still negatively correlated with reproductive success (probability of reproducing for males and females, respectively: 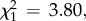 *p* = 0.05; 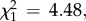 *p* = 0.03; number of children for males and females, respectively: 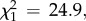 *p* < 0.001; 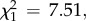 *p* = 0.006). Increased reproductive success of firstborns could also result from other mechanisms, such as parental control of the marriage order to favour earlier-borns. Our study therefore highlights the need for detailed studies of the processes underlying differential reproduction among siblings.

Although no previous studies exist in addition to the current one to document sibling fitness effects across lifetime of individuals, it is likely that the magnitude of the elder sibling effects varies across different economic systems, demographic settings or rules of inheritance. For instance, a recent paper by Gibson & Gurmu [44] suggested that within a population, the level of competition and thereby the effect of siblings was highly dependent upon the inheritance system and the presence of wealth that is not divisible. Another recent study in an Ethiopian population [55], also highlighted that the level of sibling competition was negatively correlated with the level of resources.

Our results highlight that siblings can have opposing effects on each other's fitness at different life stages, but the relative importance of positive effects during childhood on sibling survival or negative effects in adulthood on sibling reproductive success may also vary between societies or according to individual SES, depending on the importance of survival versus mating or reproductive success (or offspring quantity versus quality) in determining fitness. Thus, the optimal strategy is likely to vary according to the amount of help required to raise one's offspring successfully to adulthood, and according to the local level of mating competition. Our results thus stress the importance of considering sibling effects across the lifetime of individuals in both future theoretical and empirical studies, in order to increase our understanding of optimal reproductive scheduling and helping behaviour in given ecological settings.

Our findings have important implications, firstly for research on the evolution of optimal family size and the trade-off between offspring quantity and quality. Theoretical and empirical studies on fitness maximization in humans usually focus on optimal offspring count and on the factors influencing it [56,57]. However, in line with theoretical work [48], our study shows that considering detailed sibling configuration (i.e. intra and inter-sex birth order) is important because it influences sibling relationships. This approach is also relevant for other species; for instance, in the barn swallow (*Hirundo rustica*), the level of competition is not only dependent on offspring count, but also on brood sex-ratio [9]. Second, our study also points out the need for studying the implications and determinants of family configuration at each step of family dynamics, and in particular dispersal timing and distance. Dispersal behaviour is both the consequence of sibling interactions, and one of the factors modifying family configuration in humans [58] as well as in other species [59].

Overall, in line with a recent review of the importance of juvenile help for family evolution [18], our results suggest that elder siblings could make a beneficial contribution (e.g. by resource acquisition or by caring behaviour) towards younger sibling survival during childhood. Therefore, even if the mechanism is not known, the presence of elder siblings should be taken into account in studies of offspring quantity–quality trade-offs in the family. Moreover, our results emphasize the importance of considering all life stages in order to evaluate lifetime outcomes and trade-offs, before concluding about the selection pressures acting on sibling interactions and family evolution. The interpretation of such results could be refined by considering kin selection mechanisms. From a parental perspective, whether limiting the competition between siblings is beneficial or not depends on its consequences on overall sibship fitness. Sibling rivalry and parent–offspring conflict are thereby closely interlinked. Similarly, sex-specific negative effects between siblings can be due to direct competition but can also result from parental sex-ratio manipulation. Further studies should therefore evaluate inclusive fitness outcomes.
